# A 28-Year Multicenter Cohort Study of Nontuberculous Mycobacterial Lymphadenitis in Children, Spain

**DOI:** 10.3201/eid3103.241254

**Published:** 2025-03

**Authors:** Aina Martínez-Planas, Fernando Baquero-Artigao, Ana Méndez-Echevarría, Teresa Del Rosal, Paula Rodríguez-Molino, Carlos Toro-Rueda, Matilde Bustillo-Alonso, Miguel Lafuente, Anna Canet, Ángela Manzanares, Alfredo Tagarro, Francisco José Sanz-Santaeufemia, Sara Guillén-Martín, María José Cilleruelo, Lola Falcón-Neyra, Begoña Santiago, Elena Rincón, Miguel Lillo, Antoni Soriano-Arandes, Luigi Sedda, Clàudia Fortuny, Manuel Monsonís, Julián González-Martín, Marc Tebruegge, Antoni Noguera-Julian, Spanish Pediatric Tb Research Network, The European Nontuberculous Mycobacterial Lymphadenitis In Children Study

**Affiliations:** Institut de Recerca Pediàtrica Sant Joan de Déu, Barcelona, Spain (A. Martínez-Planas, C. Fortuny, M. Monsonís, A. Noguera-Julian); La Paz Research Institute, Madrid, Spain (F. Baquero-Artigao, A. Méndez-Echevarría, T. Del Rosal, P. Rodríguez-Molino); Universidad Autónoma de Madrid, Madrid (F. Baquero-Artigao, A. Méndez-Echevarría, T. Del Rosal, P. Rodríguez-Molino); Hospital La Paz, Madrid (F. Baquero-Artigao, A. Méndez-Echevarría, T. Del Rosal, P. Rodríguez-Molino, C. Toro-Rueda); Centro de Investigación Biomédica en Red en Enfermedades Infecciosas, Madrid (F. Baquero-Artigao, A. Méndez-Echevarría, P. Rodríguez-Molino, B. Santiago, J. González-Martín); Centro de Investigación Biomédica en Red en Enfermedades Raras, Madrid (T. Del Rosal); Hospital Universitario Miguel Servet, Zaragoza, Spain (M. Bustillo-Alonso, M. Lafuente); Hospital Universitario 12 de Octubre, Madrid, Spain (A. Canet, A. Manzanares); Instituto de Investigación 12 de Octubre, Madrid (A. Tagarro); Hospital Universitario Infanta Sofía, Madrid (A. Tagarro); Universidad Europea de Madrid, Madrid (A. Tagarro); Hospital Universitario Niño Jesús, Madrid (F.J. Sanz-Santaeufemia); Hospital Universitario de Getafe, Getafe, Spain (S. Guillén-Martín); Hospital Universitario Puerta de Hierro, Majadahonda, Spain (M.J. Cilleruelo); Hospital Virgen del Rocío, Seville, Spain (L. Falcón-Neyra); Instituto de Investigación Sanitaria Gregorio Marañón, Madrid (B. Santiago); Hospital Gregorio Marañón, Madrid (B. Santiago, E. Rincón); Hospital General Universitario de Albacete, Albacete, Spain (M. Lillo); Hospital Vall d'Hebron, Barcelona, Spain (A. Soriano-Arandes); Lancaster University, Lancaster, UK (L. Sedda); Centro de Investigación Biomédica en Red en Epidemiología y Salud Pública, Madrid (C. Fortuny, A. Noguera-Julian); Universitat de Barcelona, Barcelona, Spain (C. Fortuny, A. Noguera-Julian); Hospital Clínic, Barcelona (J. González-Martín); Austrian Reference Centre for Childhood Tuberculosis, Vienna, Austria (M. Tebruegge); Vienna Healthcare Group, Vienna (M. Tebruegge); Royal Children’s Hospital Melbourne, University of Melbourne, Melbourne, Victoria, Australia (M. Tebruegge); UCL Great Ormond Street Institute of Child Health, London, UK (M. Tebruegge).

**Keywords:** tuberculosis and other mycobacteria, bacteria, lymphadenitis, interferon-gamma release assay, Mycobacterium avium complex, Mycobacterium lentiflavum, nontuberculous mycobacteria, seasonality, tuberculin skin test, Spain

## Abstract

We describe the epidemiology, diagnosis, and management of nontuberculous mycobacterial lymphadenitis cases detailed in a 28-year (1996–2023) multicenter cohort from Spain. The case numbers remained stable during the initial prospective phase (2013–2020), but a sharp decline was observed during 2021–2022. Disease onset occurred during spring or June in 45.9% of cases. *Mycobacterium avium* complex (43.1%) and *M*. *lentiflavum* (39.9%) were the most common species detected. *M*. *lentiflavum* affected mostly younger children from central Spain. The most common treatment strategy was complete surgical resection with (n = 80) or without (n = 88) antimicrobial drug treatment, followed by antimicrobial drugs alone (n = 76). Facial palsy developed in 10.4% of surgical cases. Adverse events because of antimicrobial drugs were uncommon. New fistula formation during follow-up occurred more in children managed with observation alone than in those treated with antimicrobial drugs alone (relative risk 2.7 [95% CI 1.3–5.3]; p = 0.014).

Nontuberculous mycobacteria (NTM) are ubiquitous in soil, water, foodstuffs, and domestic and wild animals. There are >190 known species of NTM (*1*). Cervicofacial lymphadenitis is the most common clinical manifestation of NTM infection in young immunocompetent children (*2*). Cervicofacial lymphadenitis typically manifests with a nontender neck mass that progressively becomes violaceous and fluctuant and often fistulizes (*1*,*3*).

*Mycobacterium avium* complex (MAC) is reported as the most common causative species of NTM lymphadenitis across various geographic locations, accounting for 70%–80% of cases, followed by *M*. *malmoense*, *M*. *hemophilum*, and *M*. *kansasii* (*4*–*8*). *M*. *lentiflavum*, which is part of the *M*. *simiae* complex, is a slow-growing NTM species first described in 1996 (*9*,*10*). *M*. *lentiflavum* is typically isolated from water and soil samples but has increasingly been reported as a pathogenic NTM species in humans over the past 15 years (*4*).

In children, the sensitivity of classical microbiological methods, such as staining techniques and cultures, by using lymph node biopsies or caseum is ≈50%–60% (*11*–*13*). Molecular methods have demonstrated improved sensitivity compared with culture, ≈70%–80% in some studies, although molecular accuracy is limited by species diversity, the lack of commercially available assays and variable performance, and often inadequate sample volumes (*3*,*12*,*14*). In the absence of microbiological confirmation, the presumptive clinical diagnosis of NTM lymphadenitis remains complex and relies on the clinical manifestations, imaging findings, and tuberculosis (TB) immunodiagnostic tests. Recent systematic reviews have recommended the combined use of the tuberculin skin test (TST) and an interferon-γ release assay (IGRA), concluding that a TST+/IGRA– constellation is strongly indicative of NTM lymphadenitis (*13*,*14*), but data to support this strategy are still limited. TST has shown high specificity and positive predictive value in the diagnosis of NTM lymphadenitis in children without TB risk factors or prior bacillus Calmette-Guérin (BCG) vaccination in a country with low TB prevalence (*15*). We previously reported IGRA assay specificity rates and positive predictive values >95% in distinguishing between patients with TB and MAC lymphadenitis (*16*). However, those results cannot necessarily be extrapolated to other geographic settings, such as regions with high TB prevalence, or to other NTM species.

A recent consensus statement of the International Pediatric Otolaryngology Group did not reach an agreement on the single best treatment modality for NTM lymphadenitis (*17*). A meta-analysis published in 2015 reported the highest cure rate by using complete excision, compared with prolonged antimicrobial treatment or observation alone (*6*). However, complete excision was associated with a 10% risk for facial nerve palsy. Currently, the optimal combination of antimycobacterial drugs and treatment duration remains uncertain, as does whether antimycobacterial drug treatment confers advantages over observation alone (*18*).

This study aimed to describe the epidemiologic, clinical, and microbiological characteristics of NTM lymphadenitis in Spain over a 28-year period, to assess the diagnostic value of combined TST and IGRAs use, and to summarize the treatment strategies most used and the related outcomes. Because of the limited data on *M*. *lentiflavum*, we sought to describe any differences between *M*. *lentiflavum* and MAC lymphadenitis patients.

## Patients and Methods

### Study Design

The European nontuberculous mycobacterial lymphadenitis in children (ENSeMBLE) study is a multinational, multicenter, cross-sectional observational study comprising centers and investigators within the Spanish Network for the Study of Pediatric Tuberculosis (*19*), the Paediatric Tuberculosis Network European Trials group (*20*), and the European Nontuberculous Mycobacteria Network European Trials group. The study involves a convenience sample of patients <18 years of age at diagnosis with culture- or PCR-confirmed peripheral NTM lymphadenitis, collected retrospectively during 1996–2012 and prospectively since 2013. All diagnostic and therapeutic decisions were made independently at each center by the patient’s physician. We obtained study data exclusively from routine care and collected by using REDCap electronic data capture tools (*21*), hosted at Instituto de Investigación Sanitaria Gregorio Marañón (Madrid, Spain). Ethics approval for this study was obtained from Hospital Sant Joan de Déu (Barcelona, Spain) Ethics Committee (reference no. EPA 04–15). In the prospective study arm, informed consent from parents or legal guardians was obtained before inclusion. Only patients recruited at centers in Spain, representing >90% of patients in the ENSeMBLE study, were included in this report. In Spain, a low TB prevalence country, the pediatric TB incidence was <10 cases/100,000 persons throughout the study period. Neonatal BCG vaccination was discontinued nationwide in 1980, except in Basque Country, which continued until 2013.

### Data Collection

Clinical and epidemiologic data (age, sex, country of birth, underlying medical conditions, TB infection risk factors, and BCG vaccination history) were recorded at diagnosis by the clinical care team. We classified the clinical manifestations according to affected sites, laterality (unilateral or bilateral), lymph node size assessed clinically or by ultrasound (in centimeters), duration of illness (in weeks), and clinical stage (I, painless, firm, adherent to overlying skin, increased vascularity; II, fluctuance; III, skin changes, violaceous discoloration, thinning of the skin, parchment-like changes, shiny appearance; IV, fistulization) (*22*). We also collected details about treatment strategies (observation only, antimicrobial drugs, surgery, or a combination of treatments), total duration of follow-up after diagnosis, complications (surgical site infection, drug adverse events, new fistula formation, recurrent NTM infection, and paradoxical worsening), and sequelae at the end of follow-up (hypertrophic scar or keloid, changes in skin color, and transient or permanent facial nerve palsy).

### Immunological and Microbiological Tests

TST were performed by intradermal injection of 2 tuberculin units of purified protein derivative (Statens Serum Institut, https://en.ssi.dk), with results read after 48–72 hours. As per national guidelines, an induration of >5 mm diameter is considered positive, irrespective of prior BCG vaccination (*23*). All IGRA assays, including the QuantiFERON-TB (QFT) assays QFT Gold (used before 2007), QFT Gold-in-Tube (used during 2007–2016), and QFT Gold Plus (used since 2016) (all Cellestis, https://www.cellectis.com) and T-SPOT.*TB* (Oxford Immunotec LTD., https://www.oxfordimmunotec.com), were performed in fully-accredited diagnostic laboratories at each participating institution and interpreted according to the manufacturer’s instructions. Cultures and molecular assays for NTM were also performed at fully accredited clinical laboratories at the participating institutions or at regional reference laboratories.

### Statistical Analysis

We present categorical data as absolute numbers and proportions, continuous variables as medians and interquartile ranges (IQRs). We compared groups by using Student *t*-test or Mann-Whitney U test for continuous variables and χ^2^ tests for categorical variables. Because patients from provinces surrounding Madrid are usually referred to hospitals in Madrid, we categorized geographic origin dichotomously as central (Madrid and surrounding areas) and peripheral regions in Spain. We determined the onset of symptoms by subtracting the illness duration (available for 268 cases) from the date of microbiological diagnosis. We defined the seasons of the year as spring, March–May; summer, June–August; autumn, September–November; and winter, December–February.

We handled missing data with the complete case analysis method. We defined statistical significance as a 2-sided p value <0.05. We conducted statistical analyses by using SPSS Statistics 29.0 (IBM, https://www.ibm.com).

## Results

**Figure 1:**
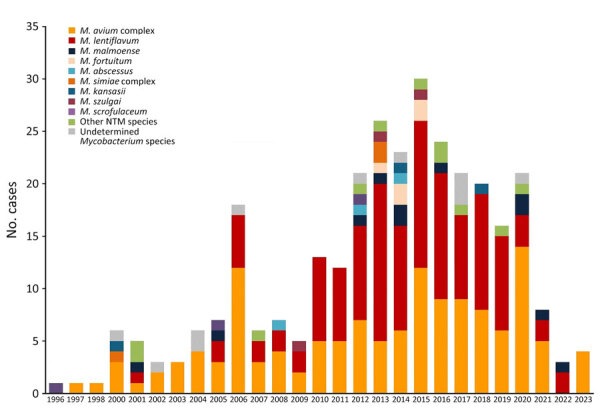
Annual case numbers of NTM lymphadenitis and the causative species in a 28-year multicenter cohort study of NTM lymphadenitis in children in Spain, 1996–2023. NTM, nontuberculous mycobacteria.

**Figure 2:**
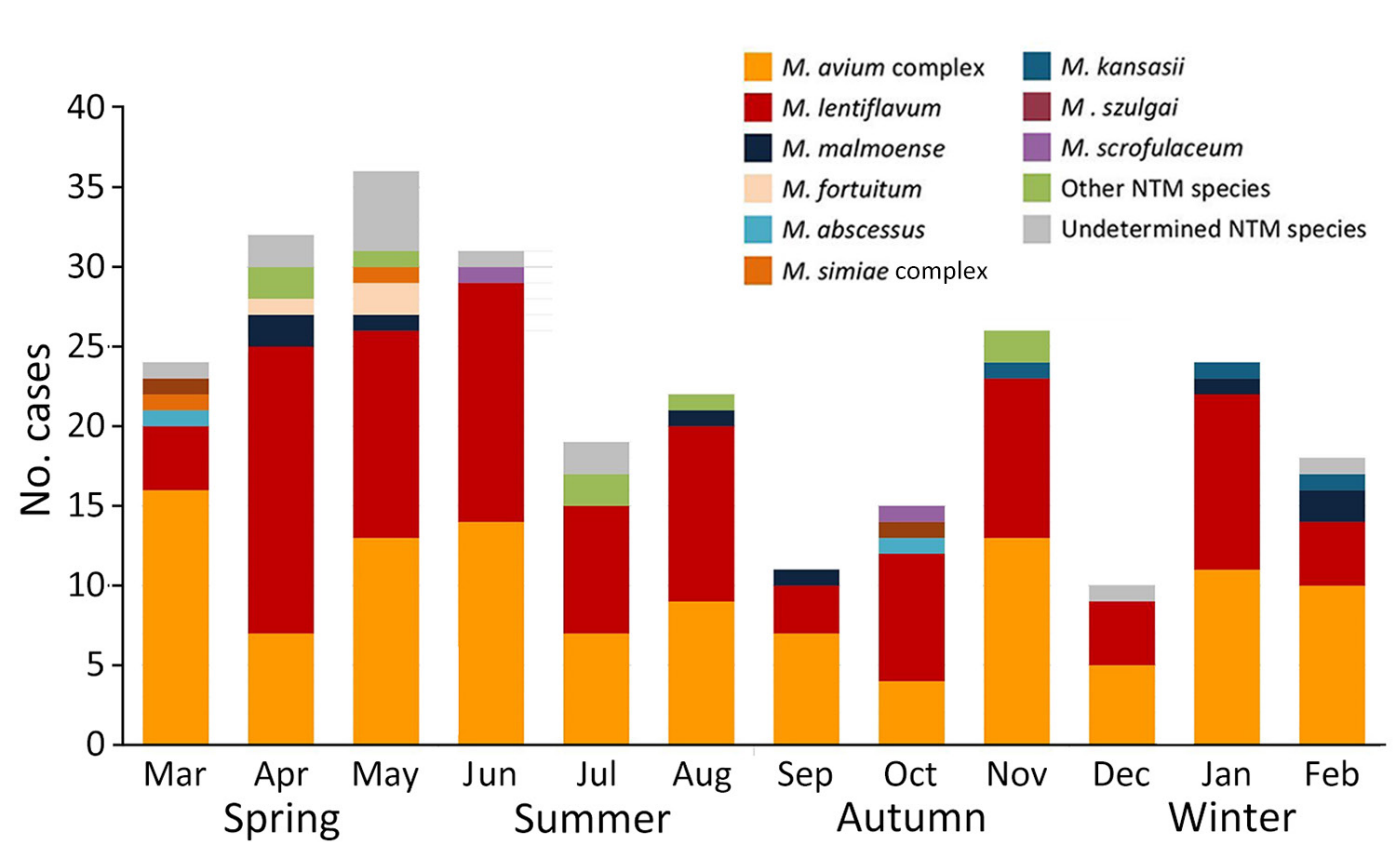
Seasonality of symptom onset, stratified by NTM species, in a 28-year multicenter cohort study of NTM lymphadenitis in children, Spain. NTM, nontuberculous mycobacteria.

By May 2023, a total of 311 case-patients (53.7% female, 46.3% male; median [IQR] age at diagnosis 2.4 [1.7–3.2] years) with microbiologically confirmed NTM lymphadenitis were contributed to the ENSeMBLE study by 33 centers in Spain from 13 of 17 administrative regions; the earliest retrospective case was discovered in 1996 (Table 1; Figure 1). Most cases (63.0%, n = 196) were contributed during the prospective phase of the study, January 2013–2023. The number of cases remained stable from 2013–2020, but a sharp decrease was observed in 2021 and 2022 (Figure 1). Symptom onset occurred during the spring months or June in almost half the patients (45.9%, n = 123) (Figure 2).

**Table 1:** Baseline characteristics, clinical manifestations, treatment, outcomes, and comparisons of MAC and *M. lentiflavum* lymphadenitis cases from a 28-year multicenter cohort study of NTM lymphadenitis in children, Spain*

Characteristics	All, n = 311	MAC, n = 134	*M*.* lentiflavum*, n = 124	p value†
Sex				
F	167 (53.7)	77 (57.5)	67 (54.0)	0.579
M	144 (46.3)	57 (42.5)	57 (46.0)	
Age, y, median (IQR)	2.4 (1.7–3.2)	2.7 (2.0–3.8)	1.9 (1.6–2.6)	<0.001
Patients <5 y of age at diagnosis	26 (8.4)	14 (10.4)	4 (3.2)	0.042
Prospective phase	196 (63.0)	78 (58.2)	88 (71.0)	0.041
Season at symptom onset‡				0.338
Spring	102 (38.1)	39 (33.6)	42 (38.5)	
Summer	58 (21.6)	22 (19.0)	28 (25.7)	
Autumn	46 (17.2)	23 (19.8)	17 (15.6)	
Winter	62 (23.1)	32 (27.6)	22 (20.2)	
Reported in central Spanish regions	189 (60.8)	52 (38.8)	107 (86.3)	<0.001
TB infection risk factors	11 (3.5)	4 (3.0)	2 (1.6)	0.685
Positive TST	168/278 (60.4)	76/118 (64.4)	62/111 (55.9)	0.186
TST induration, mm, median (IQR)	7.0 (0–10)	7.5 (4–11)	7.0 (0–10)	0.055
NTM lymphadenitis disease characteristics				
Unilateral disease	282 (90.7)	124 (92.5)	111 (89.5)	0.395
Single site disease	242 (77.8)	106 (79.1)	90 (72.6)	0.220
Symptom duration, wks, median (IQR)	4.0 (2.0–6.0)	4.0 (2.0–7.3)	3.0 (2.0–4.0)	0.001
Maximum lymph node diameter, cm, median (IQR)	3.0 (2.1–4.0)	3.0 (2.0–4.0)	3.0 (2.1–4.0)	0.558
Clinical stage§				0.926
Stage I	148 (48.6)	62 (47.3)	62 (50.8)	
Stage II	27 (8.8)	13 (9.9)	13 (10.7)	
Stage III	105 (34.4)	44 (33.6)	37 (30.3)	
Stage IV	25 (8.2)	12 (9.2)	10 (8.2)	
Affected site				
Submandibular	197 (63.3)	74 (55.2)	91 (73.4)	0.002
Superficial/deep cervical	81 (26.0)	39 (29.1)	28 (22.6)	0.247
Preauricular	44 (14.1)	13 (9.7)	25 (20.2)	0.019
Parotid	28 (9.0)	10 (7.5)	12 (9.7)	0.523
Jugulodigastric	22 (7.1)	15 (11.2)	6 (4.8)	0.062
Other¶	22 (7.1)	15 (11.2)	3 (2.4)	0.119
Treatment and outcomes				
Initial treatment strategy				0.006
Observation alone	25 (8.0)	15 (11.2)	5 (4.0)	
Antimicrobial drugs alone	76 (24.5)	22 (16.4)	35 (28.2)	
Drainage alone	6 (1.9)	2 (1.5)	2 (1.6)	
Drainage + antimicrobial drugs	29 (9.3)	14 (10.5)	10 (8.1)	
Complete resection alone	80 (25.7)	46 (34.3)	25 (20.2)	
Complete resection + antimicrobial drugs	88 (28.3)	32 (23.9)	43 (34.7)	
Not reported	7 (2.3)	3 (2.2)	4 (3.2)	
Lost to follow-up	23 (7.4)	12 (9.0)	9 (7.3)	0.618
Recurrent NTM lymphadenitis	16 (5.6)	10 (8.2)	6 (5.2)	0.257
New fistulization#	36 (12.9)	20 (18.2)	9 (8.6)	0.042
Sequelae				
None	188 (65.3)	78 (63.4)	76 (66.7)	0.600
Hypertrophic scar	52 (18.1)	25 (20.5)	15 (13.0)	0.126
Skin discoloration	27 (9.4)	13 (10.7)	12 (10.4)	0.956
Transient facial palsy	23 (8.0)	11 (9.0)	10 (8.7)	0.931
Permanent facial palsy	7 (2.4)	1 (0.8)	5 (4.3)	0.093
Frey syndrome	1 (0.3)	0 (0)	1 (0.9)	0.986

*Values are no. (%) except as indicated. Groups were compared with Student *t*-tests or Mann-Whitney U tests for continuous variables and χ^2^ tests for categorical variables. MAC, *Mycobacterium avium* complex; NTM, nontuberculous mycobacteria; TB, tuberculosis; TST, tuberculin skin test. †Comparison between MAC and *M. lentiflavum* cases. ‡Available in 268 cases in the whole cohort (116 MAC and 109 *M. lentiflavum* cases). §Available in 305 cases in the whole cohort (131 MAC and 122 *M. lentiflavum* cases). ¶Includes posterior cervical (n = 6), occipital (n = 5), postauricular (n = 4), inguinal (n = 3), axillar (n = 2), and supraclavicular (n = 2). #Excludes patients with clinical stage IV (fistulization) at initial examination.

Most children were born in Spain (95.8%, n = 298) and were not BCG-vaccinated (96.1%, n = 299). Four (1.3%) patients had underlying medical conditions (Appendix Table 1); no underlying medical conditions were discovered in the remaining children. Risk factors for TB infection were identified in 3.5% (n = 11) of children, including contact with a smear-positive TB patient (n = 5) and birth in or travel to a high TB prevalence country (n = 6). 

NTM disease predominantly affected the cervicofacial region (99.0%, n = 308). Only 2 patients had lymphadenitis in the axillary region and 3 patients had lymphadenitis in the inguinal region. In most cases, lymphadenitis only occurred at a single site (77.8%, n = 242) and was unilateral (90.7%, n = 282) (Table 1). The most affected site was the submandibular region (63.3%, n = 197). 

At initial examination, the median (IQR) duration of symptoms was 4 weeks (2–6, data available in n = 268 cases), and the maximum lymph node diameter was 3.0 cm (2.1–4.0, n = 108). Almost half the cases were in Penn Stage I upon initial examination (Table 1). Similar results were observed when the retrospective and prospective phases of the study were analyzed separately (Appendix Tables 2, 3).

### Causative NTM Species

**Figure 3:**
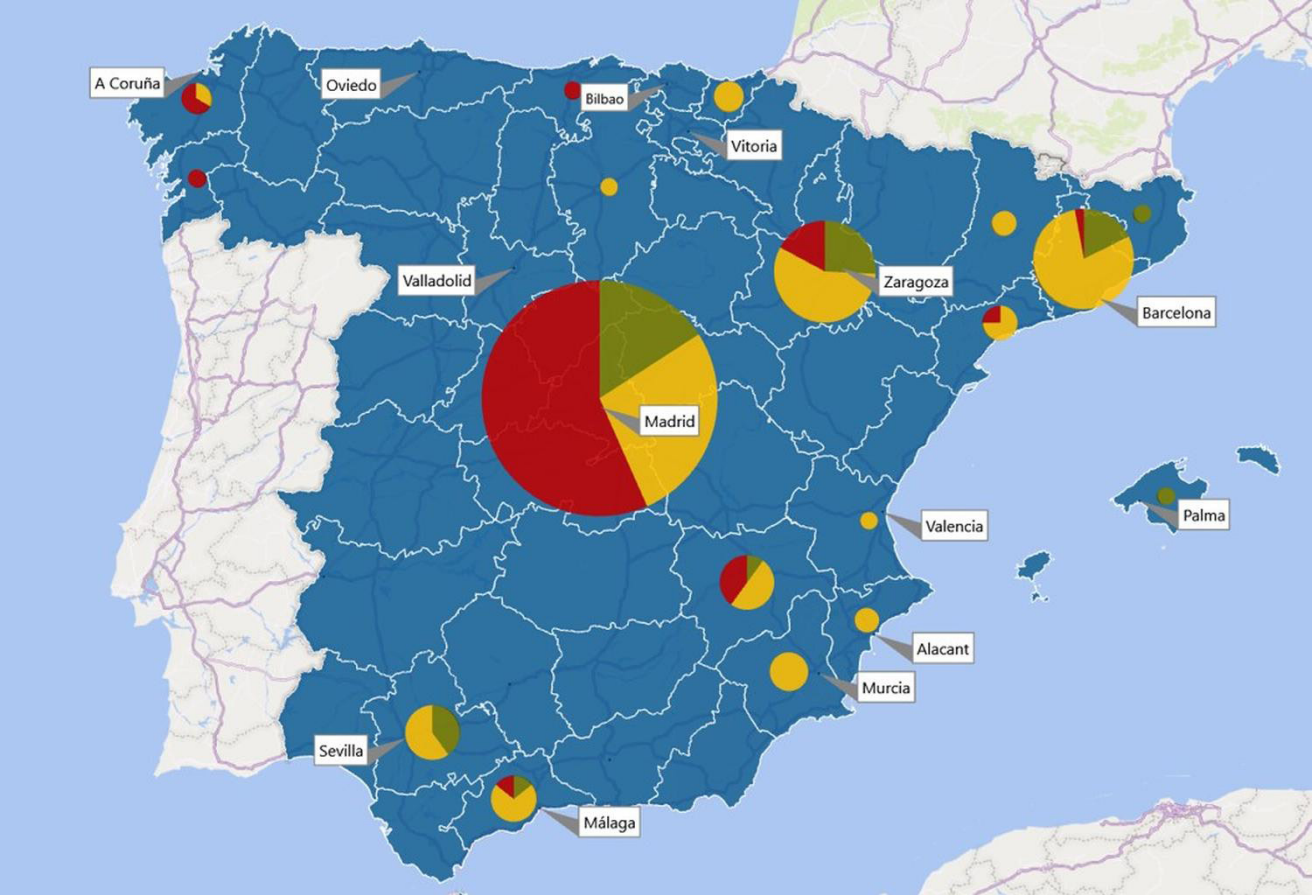
Geographic distribution of nontuberculous mycobacterial lymphadenitis cases in Spain. The pie charts show the species in each region: yellow, *Mycobacterium avium* complex; red, *Mycobacterium lentiflavum*; green, all other species. The size of each pie chart is proportional to the number of cases reported in the respective region.

Microbiological confirmation was obtained at the site of disease (lymph node biopsy, fine needle aspiration, or discharge fluid) in all cases. Mycobacterial cultures were positive in 96.8% (n = 300) of cases and molecular assays were positive in 48.8% (n = 40) of cases when performed; in patients in whom both culture and a molecular test were performed, both techniques yielded a positive result in 35.8% (n = 29) of the time (Appendix Table 4). The most frequently identified NTM species were MAC (43.1%, n = 134) and *M*. *lentiflavum* (39.9%, n = 124), followed by *M*. *malmoense* (3.5%, n = 11); *M*. *fortuitum* (1.6%, n = 5); *M*. *abscessus*, *M*. *kansasii*, *M. scrofulaceum*, *M. simiae* complex, and *M. szulgai* (1.0%, n = 3 each); *M. interjectum* (0.6%, n = 2); and *M*. *chelonae*, *M*. *colombiense*, *M*. *mageritense*, *M*. *marinum*, *M*. *mucogenicum*, *M*. *triplex*, and *M*. *xenopi* (0.3%, n = 1 each). In 13 (4.2%) patients, the NTM species could not be determined. NTM species are widely distributed geographically in Spain (Figure 3).

### Immunologic Tests

TSTs were performed in 89.4% (n = 278) of cases and reported positive in 168 cases, corresponding to a test sensitivity of 60.4% (95% CI 54.4%–66.2%) (Table 1). IGRA assays were performed in 44.4% (n = 138) of cases (QFT assays only, n = 111; T-SPOT.*TB* only, n = 11; both assays, n = 16). Of those cases, 89.9% (n = 124) were negative, 6.5% (n = 9) were positive, and 3.6% (n = 5) had an indeterminate test result. Overall, 138 cases were tested with both the TST and an IGRA assay, 67.4% (n = 93) had a TST+/IGRA– constellation (Appendix Table 5). Among children with a positive IGRA result (Appendix Table 6), epidemiologic risk factors for TB infection were identified in 3 patients. In addition, 4 patients with positive results underwent repeat assays that yielded negative results. *M*. *kansasii* (n = 3), *M*. *szulgai* (n = 3), and *M*. *marinum* (n = 1) are all NTM species that are known to express the ESAT-6 protein and potentially cause false-positive IGRA results (*24*,*25*). An IGRA assay was performed in 1 case caused by *M*. *szulgai* and was positive.

### Treatment and Outcomes

Various treatment strategies were used with differing outcomes in cases of NTM lymphadenitis (Table 1). The most common treatment strategy consisted of complete surgical resection with (n = 88) or without (n = 80) antimycobacterial antimicrobial drugs, followed by antimicrobial drug therapy alone (n = 76). Pyogenic surgical site superinfection occurred in 3.4% (n = 7) of cases who underwent surgery. Overall, 62.1% (n = 193) patients initially received antimicrobial drugs for a median (IQR) time of 16 (8–24) weeks; the most used regimens were a macrolide combined with ciprofloxacin (49.2%, n = 95), rifampin (10.4%, n = 20), ethambutol (9.3%, n = 18) or rifabutin (5.7%, n = 11). Of the cases treated with antimicrobial drugs, 8.3% (n = 16) were treated with clarithromycin only, 7.3% (n = 14) received 3-drug regimens, and 1.6% (n = 3) received 4-drug regimens (Appendix Table 7). No cases of paradoxical worsening were reported. Adverse events because of antimicrobial drugs were uncommon (gastrointestinal symptoms n = 3; neutropenia n = 3; hearing loss n = 1; lethargy n = 1).

Follow-up data were available for 92.6% (n = 288) of cases (median [IQR] follow-up time from diagnosis 0.6 [0.3–1.0] years) (Table 2). Unplanned treatment during follow-up was performed in 18.4% (n = 53) cases and included surgery with (n = 3) or without (n = 44) antimicrobial drugs or antimicrobial drugs alone (n = 6) (Appendix Table 7). Among 280 cases with Penn clinical stages I to III at initial examination, fistula formation occurred in 12.9% (n = 36) of cases. Of those cases, children who were managed with observation alone had a significantly higher risk for fistula formation than those treated with antimicrobial drugs alone (45.0% [n = 9] vs. 16.4% [n = 10]; relative risk 2.7 [95% CI 1.3–5.3]; p = 0.014). Recurrent NTM lymphadenitis after resolution of symptoms and signs of the initial clinical manifestation occurred in 5.6% (n = 16) children.

**Table 2:** Complication details during follow-up by initial therapeutic strategy at initial examination of patients from a 28-year multicenter cohort study of NTM lymphadenitis in children, Spain*

Characteristics	Observation	Antimicrobial drugs alone	Drainage alone	Drainage + antimicrobial drugs	Complete resection alone	Complete resection + antimicrobial drugs	p value
Need for unplanned treatment	1/25 (4.0)	20/72 (27.8)	3/6 (50.0)	13/29 (44.8)	4/77 (5.2)	11/88 (12.5)	0.033
New fistula formation†	9/20 (40.0)	10/61 (16.4)	2/5 (40.0)	3/20 (15.0)	2/71 (2.8)	10/82 (12.2)	0.002
Recurrent NTM infection	0/21 (0)	1/67 (1.5)	2/6 (33.3)	0/27 (0)	2/76 (2.6)	10/87 (11.5)	0.020
Sequelae							
None	15/21 (71.4)	48/67 (71.6)	5/6 (83.3)	13/27 (48.1)	55/76 (72.4)	48/87 (55.2)	0.073
Hypertrophic scar	2/21 (9.5)	11/67 (16.4)	1/6 (16.7)	8/27 (29.6)	9/76 (11.8)	21/87 (24.1)	0.244
Skin discoloration	4/21 (19.0)	8/67 (11.9)	0/6 (0)	3/27 (11.1)	5/76 (6.6)	7/87 (8.0)	0.122
Transient facial palsy	0/21 (0)	2/67 (3.0)	0/6 (0)	4/27 (14.8)	8/76 (10.5)	9/87 (10.3)	0.025
Permanent facial palsy	0/21 (0)	1/67 (1.5)	0/6 (0)	0/27 (0)	1/76 (1.3)	5/87 (5.7)	0.089
Facial palsy	0/21 (0)	3/67 (4.5)‡	0/6 (0)	4/27 (14.8)	9/76 (11.8)	14/87 (16.1)	0.004

*Values are no. patients/total no. evaluated (%). NTM, nontuberculous mycobacteria †Excludes patients with clinical stage grade IV (with fistula) at initial examination. ‡All 3 patients were initially managed only with antimycobacterial antimicrobial drugs, but subsequently underwent surgery; facial palsy developed only after the surgical intervention. Patients with preexisting medical conditions at diagnosis (n = 4) were excluded from this analysis. Groups were compared by using chi-square test

At the last available follow-up, 65.3% (n = 188) children were reported to have no sequelae. Among children with sequelae, the most common findings were hypertrophic scar or keloid (18.1%, n = 52) and skin discoloration (9.4%, n = 27). Transient facial palsy occurred in 8.0% (n = 23) of cases, and permanent facial palsy occurred in 2.4% (n = 7) of cases. All case-patients had undergone surgery either when diagnosed or during follow-up. The affected sites were submandibular (n = 20), parotid (n = 6), superficial or deep cervical (n = 6), jugulodigastric (n = 5), and preauricular (n = 4). Frey syndrome developed after excisional surgery in a patient with *M*. *lentiflavum* cervical and preauricular lymphadenitis.

After excluding patients with underlying medical conditions, patients treated with antimicrobial drugs alone or drainage (with or without antimicrobial drugs) more often required unplanned treatment, which included surgery in most cases (Table 2). No significant differences between initial treatment strategies were observed regarding aesthetic sequelae, but facial palsy was significantly more common among patients who had undergone surgery at diagnosis (Table 2). Further detailed analyses revealed no other risk factors associated with the development of sequelae (Appendix Table 8). A subgroup analysis including only children with clinical stage I lymphadenitis at initial clinical examination showed similar results (Appendix Table 9).

### Comparison between Dominant Species MAC and *M*. *lentiflavum*

Children with lymphadenitis caused by *M*. *lentiflavum* were younger at initial clinical examination, more often reported in the prospective phase of the study, and more common in central Spain (86.3%, n = 107 isolates), whereas MAC was more prevalent in the peripheral regions (61.2%, n = 82 isolates) (Table 1; Figure 3). The duration of symptoms before initial examination was shorter in *M*. *lentiflavum* cases, and submandibular and preauricular sites tended to be more commonly affected (Table 1). Differences were observed in the initial treatment strategies and the rate of new fistula formation, which was more common in MAC cases.

## Discussion

This large study of children with microbiologically confirmed NTM lymphadenitis resulted from collaboration between 3 large mycobacterial research networks. Because of the participation of 33 tertiary and quaternary units providing healthcare for children with NTM infections distributed widely across Spain, we were able to identify several epidemiologic trends. First, the number of NTM lymphadenitis cases in the prospective phase initially remained stable until 2020, followed by a sharp decline coinciding with the COVID-19 pandemic, a trend observed in many other childhood infections (*26*). Because our study was on the basis of a convenience sample, we were not able to calculate incidence rates, but previous studies from the Netherlands, Germany, Wisconsin (USA), and Australia have reported incidences of 0.8–3.3 cases/100,000 children, although each study used different inclusion criteria (*2*,*27*–*29*). Of note, the ENSeMBLE study was deliberately designed to have stringent entry criteria that included the presence of microbiological confirmation, which led to high validity of our data but also resulted in a smaller cohort than if cases solely identified on clinical grounds were included. Second, our data confirm the observation that case numbers of NTM lymphadenitis typically peak in spring in countries with moderate climate, a phenomenon that was first described by a single-center study from Australia (*3*). Third, geographic differences in the distribution of causative NTM species across the country were observed; *M*. *lentiflavum* was responsible for most cases in the central regions of Spain, whereas MAC predominated in almost all other regions.

In our cohort, *M*. *lentiflavum* was almost as common as MAC, which was the predominant agent in almost all previous studies on NTM lymphadenitis (*3*,*27*–*29*). Until the early 21st Century, *M*. *lentiflavum* was rarely reported as a causative agent of disease in humans. A meta-analysis in 2015 identified only 1 case (of 1,274) of lymphadenitis caused by *M*. *lentiflavum* (*6*). Nevertheless, this NTM species was described as an emerging pathogen in several small case series of NTM lymphadenitis in southern Europe over the past decade (*4*,*30*–*32*) and in cystic fibrosis patients (*33*–*35*). It was hypothesized that *M*. *lentiflavum* emergence might be because of improvements in identification techniques such as molecular tests and sequencing, rather than the result of an ecologic evolution (*30*). Of note, when we compared the 2 most prevalent species in our study, *M*. *lentiflavum* tended to affect younger patients, mainly occurred in the center of the country, had a faster disease course, and predominantly involved submandibular and preauricular lymph nodes compared with MAC. Our findings suggest that a combination of bacterial, host, and environmental factors might play a role in the recent emergence of *M*. *lentiflavum* (*36*).

In this study, IGRA assays yielded negative results in almost 90% of cases, but a TST+/IGRA– constellation was only observed in two thirds of the cases that underwent both tests. Our results support the dual immunodiagnostic strategy previously reported (*1*,*13*,*14*), but also highlight several limitations. First, we did not include an uninfected control group and therefore could not calculate specificity rates. Second, IGRA assays are not universally available, particularly in low-resource settings where TB lymphadenitis plays a greater role. Third, positive IGRA results were observed in 9 patients in our cohort and were because of different reasons: infection by *M*. *szulgai*, an NTM species known to express ESAT-6 (*24*,*25*); probable concomitant TB infection (in children with epidemiologic risk factors); and false-positive IGRA results with borderline interferon-γ responses that reverted to negative upon repeat testing. Finally, TST results were negative in 39.6% (n = 110) of cases in which this test was performed. This finding aligns with data from previous studies, which have reported TST results to be negative in 30%–50% of patients with NTM lymphadenitis (*1*). Nevertheless, in the absence of microbiological confirmation in a child with compatible symptoms and signs, a TST+/IGRA– result constellation supports the diagnosis of NTM lymphadenitis. However, such findings should be considered together with the results of other investigations as part of a comprehensive diagnostic work-up.

Our study was observational, with small sample sizes for some treatment options and a risk of confounding bias; therefore, the treatment and outcome data must be interpreted with caution. In contrast with 2 studies from the same group, we found that antimycobacterial treatment was generally well tolerated and that adverse events were rare and typically short-lived (*37*,*38*). In comparison, excisional surgery was associated with a substantial risk for facial nerve palsy, of which 8.0% of cases were transient and 2.4% permanent. Those data are similar to data from a previous meta-analysis documenting 7.6% transient facial nerve palsy and 2.1% permanent facial nerve palsy (*6*). As previously reported, our data also confirm that drainage alone is an inadequate management option, because most patients require further interventions (*39*–*41*). Furthermore, in children without fistula at initial clinical examination, those managed with observation alone had an almost 3-fold higher risk for developing a fistula during the disease course compared with children treated with antimycobacterial antimicrobial drugs alone. Ultimately, treatment decisions should consider diagnostic certainty, location and extent of the disease, local surgical experience, and parental preferences (*1*,*17*,*18*).

Our study is limited as an ambispective observational design, inevitably resulting in some data not being available. During the prospective phase of the study, two thirds of the total cases were reported, likely because of ascertainment bias. Also, we did not collect data on acid-fast staining, culture media, or the molecular assays used across different sites, some assays being noncommercial in-house assays. Although <50% of cases had both TST and IGRA testing completed, the cohort size enabled us to produce meaningful data. Randomized trials would be beneficial to clarify the optimal therapeutic strategy for NTM lymphadenitis, ideally with stratification according to clinical stage at initial clinical examination.

In conclusion, in this 28-year national cohort of microbiologically confirmed NTM lymphadenitis, *M*. *lentiflavum* emerged as a major causative species. Temporal analyses revealed seasonal peaks in spring and troughs in autumn. Our data support the combined use of TST and an IGRA assay in the diagnostic workup of protracted cervical lymphadenitis in young children pending microbiological results, although positive IGRA results can occur and require careful interpretation. Complete surgical resection was associated with a substantial risk for facial nerve palsy. Observation alone was associated with a higher risk for new fistula formation than treatment with antimycobacterial antimicrobial drugs, which were overall well tolerated. 

Appendix Additional information about a 28-year multi-center cohort study of nontuberculous mycobacterial lymphadenitis in children, Spain.
