# Supplementary material for: A 28-Year Multicenter Cohort Study of Nontuberculous Mycobacterial Lymphadenitis in Children, Spain
Source: Emerg Infect Dis. 2025 Mar;31(3):536–46. doi: 10.3201/eid3103.241254 (PMC11878309; doi:10.3201/eid3103.241254)
Supplement: Appendix — Additional information about a 28-year multi-center cohort study of nontuberculous mycobacterial lymphadenitis in children, Spain. [file 24-1254-Techapp-s1.pdf]

Article DOI: https://doi.org/10.3201/eid3103.241254

EID cannot ensure accessibility for supplementary materials supplied by authors. Readers who have difficulty accessing supplementary content should contact the authors for assistance.

A 28-Year Multicenter Cohort Study of Nontuberculous Mycobacterial Lymphadenitis in Children, Spain

Appendix

Appendix Table 1. Details of the 4 patients with pre-existing medical conditions at diagnosis.

Case no.	Sex, age (years)	Pre-existing disease	NTM species	Clinical presentation	Treatment and outcomes
1	Male, 4.2	MSMD	*M. fortuitum*	12-wk grade III lymphadenitis affecting the axillary, inguinal, submandibular, occipitial and cervical regions	Azithromycin + ciprofloxacin + co-trimoxazole for 18mo until the patient successfully underwent allogenic bone marrow transplant; no complications or sequelae from NTM disease
2	Female, 2.9	Myeloblastic leukemia (cured 18 mo earlier)	MAC	6-wk unilateral submandibular lymphadenitis (3.5cm diameter)	Total surgical resection; no complications or sequelae
3	Male, 2.8	NEMO deficiency syndrome	*M. lentiflavum*	2-wk grade IV unilateral lymphadenitis affecting the preauricular and submandibular sites (2cm diameter)	Initial surgical resection; submandibular lymphadenitis recurred 9mo later and required subsequent surgery
4	Male, 2.3	Chronic granulomatous disease	*M. simiae* complex	3-wk grade III lymphadenitis affecting the cervical and postauricular sites	Clarithromycin + ciprofloxacin, which the parents discontinued after 2wk when spontaneous fistulization occurred

MAC, *Mycobacterium avium* complex; mo, month; MSMD, mendelian susceptibility to mycobacterial diseases; NEMO, nuclear factor-kappa B essential modulator; NTM, non-tuberculous mycobacteria; wk, week.

Appendix Table 2. Baseline characteristics and details about clinical presentation in patients included in the restrospective phase (1996–2012), and comparisons between patients with MAC lymphadenitis and those with *M. lentiflavum* lymphadenitis. Data are expressed as numbers (percentages) or medians (IQR). Groups were compared with Student's t tests or Mann-Whitney *U* tests for continuous variables and chi-square tests for categorical variables.

Characteristic	All (n = 115)	MAC (n = 56)	*M. lentiflavum* (n = 38)	*p*-value*
Female gender	58 (50.4)	31(55.3)	18 (47.4)	0.447
Age at diagnosis (years)	2.4 (1.7–3.0)	2.7 (2.0–3.8)	2.0 (1.6–2.7)	0.002
Season at symptom onset†				0.276
Spring	18 (20.2)	16 (34.8)	7 (26.0)	
Summer	29 (32.6)	12 (26.1)	9 (33.3)	
Autumn	25 (28.1)	8 (17.4)	6 (22.2)	
Winter	17 (19.1)	10 (21.7)	5 (18.5)	
Reported in central Spanish regions	79 (68.7)	32 (57.1)	34 (89.5)	<0.001
TB infection risk factors present	3 (2.6)	2 (3.6)	0	0.147
NTM lymphadenitis disease characteristics				
Unilateral disease	107 (93.0)	52 (92.9)	34 (89.5)	0.567
Single site disease	83 (72.2)	42 (75.0)	22 (57.9)	0.081
Duration of symptoms (weeks)	4.0 (2.0–6.0)	4.0 (2.0–8.0)	3.0 (2.0–5.5)	0.406
Maximum diameter of lymph node (cm)	2.8 (2.0–3.0)	3.0 (2.0–3.0)	2.0 (1.9–2.1)	0.076
Clinical stage (22) ‡				0.323
Stage I	55 (49.5)	30 (55.65)	16 (43.2)	
Stage II	11 (9.9)	6 (11.1)	5 (13.5)	
Stage III	36 (32.4)	16 (29.6)	11 (29.8)	
Stage IV	9 (8.2)	2 (3.7)	5 (13.5)	
Affected site				

Characteristic	All (n = 115)	MAC (n = 56)	*M. lentiflavum* (n = 38)	*p*-value*
Submandibular	77 (67.0)	37 (66.1)	26 (68.4)	0.812
Superficial/deep cervical	35 (30.4)	16 (28.6)	14 (36.8)	0.399
Preauricular	21 (18.3)	7 (12.5)	11 (28.9)	0.047
Parotid	8 (7.0)	2 (3.6)	5 (13.2)	0.117
Jugulodigastric	5 (4.3)	3 (5.4)	2 (5.3)	0.984
Other	6 (5.2)	5 (8.9)	0	0.079

MAC, *Mycobacterium avium* complex; NTM, non-tuberculous mycobacteria.
*Comparison between MAC and *M. lentiflavum* cases.
†Available in 89 cases (including 46 MAC and 27 *M. lentiflavum* cases).
‡Available in 111 cases (including 54 MAC and 37 *M. lentiflavum* cases).

Appendix Table 3. Baseline characteristics and details about clinical presentation in patients included in the prospective phase (2013–2023), and comparisons between patients with MAC lymphadenitis and those with *M. lentiflavum* lymphadenitis. Data are expressed as numbers (percentages) or medians (IQR). Groups were compared with Student's t tests or Mann-Whitney *U* tests for continuous variables and chi-square tests for categorical variables.

Characteristics	All (n = 196)	MAC (n = 78)	*M. lentiflavum* (n = 86)	*p*-value*
Female sex	109 (55.6)	46 (59.0)	49 (57.0)	0.796
Age at diagnosis (years)	2.4 (1.7–3.4)	2.7 (1.9–3.8)	1.9 (1.6–2.6)	<0.001
Season at symptom onset†				0.573
Spring	63 (35.2)	20 (28.6)	28 (34.1)	
Summer	47 (26.2)	18 (25.6)	25 (30.5)	
Autumn	35 (19.6)	16 (22.9)	15 (18.3)	
Winter	34 (19.0)	16 (22.9)	14 (17.1)	
Reported in central Spanish regions	110 (56.1)	20 (25.6)	73 (84.9)	<0.001
TB infection risk factors present	8 (4.1)	2 (2.6)	2 (2.6)	1.000
NTM lymphadenitis disease characteristics				
Unilateral disease	177 (90.3)	72 (92.3)	77 (89.5)	0.538
Single site disease	159 (81.1)	64 (82.1)	68 (79.1)	0.630
Duration of symptoms (weeks)	3.0 (2.0–5.0)	4.0 (2.0–6.8)	3.0 (2.0–4.0)	0.002
Maximum diameter of lymph node (cm)	3.0 (2.3–4.0)	3.0 (2.3–4.0)	3.0 (2.1–4.0)	0.815
Clinical stage (*22*)‡				0.269
Stage I	93 (47.9)	32 (41.6)	46 (54.1)	
Stage II	16 (8.2)	7 (9.0)	8 (9.4)	
Stage III	69 (35.7)	28 (36.4)	26 (30.6)	
Stage IV	16 (8.2)	10 (13.0)	5 (5.9)	
Affected site				
Submandibular	120 (61.2)	37 (47.4)	65 (75.6)	<0.001
Superficial/deep cervical	46 (23.5)	23 (29.5)	14 (16.3)	0.048
Preauricular	23 (11.7)	6 (7.7)	14 (16.3)	0.099
Parotid	20 (10.2)	8 (10.3)	7 (8.1)	0.656
Jugulodigastric	17 (8.7)	12 (15.4)	4 (4.7)	0.021
Other	16 (8.2)	9 (11.5)	5 (5.8)	0.190

*Comparison between MAC and *M. lentiflavum* cases.
†Available in 179 cases (including 70 MAC and 82 *M. lentiflavum* cases).
‡Available in 194 cases (including 77 MAC and 85 *M. lentiflavum* cases).
MAC, *Mycobacterium avium* complex; NTM, non-tuberculous mycobacteria.

Appendix Table 4. Details of the microbiological investigations according to NTM species.

Species	Positive cultures/no. of cultures performed	Positive PCR tests/no. of PCRs performed	Positive PCR tests and cultures/no. of cases in whom both tests were performed
MAC (n = 134)	132/134	17/42	15/42
M. lentiflavum (n = 124)	123/124	11/25	10/25
M. malmoense (n = 11)	11/11	0/1	0/1
M. fortuitum (n = 5)	5/5	0	0
M. abscessus (n = 3)	3/3	0	0
M. kansasii (n = 3)	3/3	0	0
M. scrofulaceum (n = 3)	3/3	0/1	0/1
M. simiae complex (n = 3)	3/3	0	0
M. szulgai (n = 3)	3/3	0	0
M. interjectum (n = 2)	2/2	0/1	0/1
M. chelonae (n = 1)	1/1	0	0
M. colombienses (n = 1)	1/1	0	0
M. mageritense (n = 1)	1/1	0	0
M. marinum (n = 1)	1/1	0	0
M. mucogenicum (n = 1)	0/1	1/1	0/1
M. triplex (n = 1)	1/1	0	0
M. xenopi (n = 1)	1/1	0	0
Undetermined NTM species (n = 13)	6/12	11/11	4/10
TOTAL	300/310	40/82	29/81

MAC, *Mycobacterium avium* complex; NTM, non-tuberculous mycobacteria; PCR, polymerase chain reaction.

Appendix Table 5. Summary of concordance and discordance between tuberculin skin test and interferon-gamma release assay test results .

Test	Positive IGRA	Negative IGRA	Indeterminate IGRA	IGRA not done	Total
Positive TST (n = 168)	7	93	5	63	168
Negative TST (n = 110)	2	30	0	78	110
TST not done (n = 33)	0	1	0	32	33
Total (n = 311)	9	124	5	173	311

IGRA, interferon-gamma release assay; TST, tuberculin skin test

Appendix Table 6. Details of the patients in whom IGRA assays yielded a positive result.

Case no.	Sex, age (y)	TB infection risk factors	NTM species	TST (mm)	IGRA assay, quantitative values*	Further testing details
1	Male, 3.2	Contact with a TB patient	MAC	17	QFT-GIT, TB: 0.68	None
2†	Male, 4.2	None	*M. fortuitum*	0	QFT-GIT, ND	None
3	Female, 5.1	Contact with a TB patient	*M. szulgai*	20	QFT-GIT, TB: 8.38	None
4	Female, 1.7	None	*M. lentiflavum*	10	QFT-Plus, ND	A subsequently repeated assay yielded a negative result
5	Female, 6.9	None	*M. lentiflavum*	7	QFT-Plus, ND	A subsequently repeated assay yielded a negative result
6	Male, 2.4	None	*M. lentiflavum*	6	QFT-Plus, TB1: 0.52 and TB2: 0.65	Subsequently repeated assays yielded an indeterminate and a negative result
7	Female, 1.9	None	*M. lentiflavum*	5	QFT-Plus, TB1: 0.7 and TB2: 0.44	A subsequently repeated assay yielded a negative result
8	Male, 12.8	Travel to a high TB burden country	*M. mucogenicum*	20	QFT-Plus, ND	None
9	Female, 8.1	None	MAC	0	T-SPOT.*TB*, ND	None

*Background-corrected TB IFN-γ concentrations for QFT assays (i.e., IFN-γ concentration in antigen-stimulated tube minus nil tube concentration, in IU/ml).

†Patient 1 in **Appendix Table 1**.

IGRA, interferon-gamma release assay; MAC, *Mycobacterium avium* complex; ND, no data; NTM, non-tuberculous mycobacteria; QFT-GIT, QuantiFERON-TB Gold In-Tube; QFT-Plus, QuantiFERON-TB Gold-Plus; TB, tuberculosis.

Appendix Table 7. Details regarding antibiotic drugs and regimens that were prescribed, both as part of the initial treatment strategy and as unplanned treatment during follow-up. Data are shown as numbers (percentages).

**Initial therapeutic strategy, n = 193**	
**Individual drugs**	**n (%)**
Clarithromycin	126 (65.3)
Ciprofloxacin	103 (53.4)
Azithromycin	65 (33.7)
Rifampin	33 (17.1)
Ethambutol	29 (15.0)
Rifabutin	14 (7.3)
Levofloxacin	2 (1.0)
Isoniazid	2 (1.0)
Co-trimoxazole	1 (0.5)
Amikacin	1 (0.5)
**Treatment regimens**	**n (%)**
Clarithromycin + ciprofloxacin	63 (32.6)
Azithromycin + ciprofloxacin	32 (16.6)
Clarithromycin monotherapy	16 (8.3)
Clarithromycin + rifampin	16 (8.3)
Clarithromycin + ethambutol	9 (4.7)
Azithromycin + ethambutol	9 (4.7)
Clarithromycin + rifabutin	7 (3.6)
Azithromycin + ethambutol + 3rd drug	5 (2.6)
Azithromycin + rifampin	4 (2.1)
Azithromycin + rifabutin	4 (2.1)
Clarithromycin + ethambutol + 3rd drug	4 (2.1)
Not reported	6 (3.1)
**Unplanned antibiotic treatment during follow-up, n = 9**	
Drugs	n, treatment notes
Clarithromycin + rifabutin	3, due to recurrent NTM lymphadenitis*
Ethambutol was added to clarithromycin	1, drainage was performed
Azithromycin + ethambutol	1, due to recurrent NTM lymphadenitis
Azithromycin + ciprofloxacin	1
Not reported	3

NTM, non-tuberculous mycobacteria
*in n = 2, a 2nd surgical procedure was additionally performed

Appendix Table 8. Comparison between patients with NTM lymphadenitis who had no sequelae at the end of follow-up and those who did. Patients that were lost to follow-up (n = 23) and those with pre-existing medical conditions at diagnosis (n = 4) were excluded from this analysis. Data are shown as number (percentage) or median (IQR). Groups were compared with Student's t tests or Mann-Whitney *U* tests for continuous variables and chi-square tests for categorical variables.

Category	**No sequelae (n = 184)**	**Any sequelae (n = 100)**	***p*-value**
**Female (%)**	96 (52.2)	62 (62.0)	0.111
**Male (%)**	88 (47.2)	38 (38.0)	
**Age at diagnosis (years)**	2.4 (1.8–3.5)	2.3 (1.7–2.9)	0.148
**Contributed from January 2013 onwards, prospective phase**	122 (64.9)	56 (56.0)	0.139
**Season of the year at symptom onset***			0.213
**Spring**	51 (32.3)	40 (45.5)	
**Summer**	39 (24.7)	18 (20.5)	
**Autumn**	30 (19.0)	15 (17.0)	
**Winter**	38 (24.0)	15 (17.0)	
**Reported in Madrid region**	115 (62.5)	61 (61.0)	0.804
**TST result - positive**	98/169 (58.0)	61/93 (65.6)	0.228
**TST induration (mm)**	0 (7–10)	7.5 (4–10)	0.789
**NTM lymphadenitis disease characteristics**			
**Unilateral disease**	169 (91.8)	91 (91.0)	0.806
**Single site disease**	146 (80.4)	75 (75.0)	0.400
**Duration of symptoms (weeks)**	4.0 (2.0–6.0)	4.0 (2.0–5.0)	0.491
**Maximum diameter (cm)**	3.0 (2.0–4.0)	3.0 (2.3–4.0)	0.307
**Clinical stage (*22*)†**			0.261
**Stage I**	89 (49.7)	48 (48.0)	
**Stage II**	20 (11.2)	5 (5.0)	
**Stage III**	57 (31.8)	40 (40.0)	
**Stage IV**	13 (7.3)	7 (7.0)	
**Affected site**			
**Submandibular**	113 (61.4)	68 (68.0)	0.270

**Superficial/deep cervical**	52 (28.3)	23 (23.0)	0.337
**Preauricular**	26 (14.1)	13 (13.0)	0.792
**Parotid**	15 (8.2)	10 (10.0)	0.600
**Jugulodigastric**	9 (4.9)	11 (11.0)	0.055
**NTM species**			0.693
**MAC**	75 (40.8)	46 (46.0)	
Mycobacterium lentiflavum	76 (41.3)	38 (38.0)	
**Other species**	33 (17.9)	16 (16.0)	
**Follow-up time (years)**	0.6 (0.3–1.0)	0.7 (0.4–1.2)	0.064

*Available in 158 and 88 patients without and with sequelae, respectively.
†Available in 179 and 100 patients without and with sequelae, respectively.
MAC, *Mycobacterium avium* complex; NTM, non-tuberculous mycobacteria.

Appendix Table 9. Details on complications during follow-up and sequelae in patients with grade I clinical stage at diagnosis only, according to the initial therapeutic strategy. Patients with pre-existing medical conditions (n = 4) were excluded from this analysis. Data are expressed as numbers (percentages). Groups were compared using chi-square tests.

Category	**Observation**	**Antibiotics**	**Drainage**	**Drainage + antibiotics**	**Complete resection**	**Complete resection + antibiotics**	***p*-value**
**Need for unplanned treatment**	1/13 (7.7)	10/39 (25.6)	0/1 (0)	2/9 (22.2)	1/37 (2.7)	9/44 (20.5)	0.529
**New fistulization***	2/9 (22.2)	7/39 (17.9)	0/1 (0)	2/8 (25.0)	2/36 (5.6)	3/44 (6.8)	0.039
**Recurrent NTM infection**	0/9 (0)	1/39 (2.6)	0/1 (0)	0/8 (0)	0/36 (0)	6/44 (13.6)	0.052
**Sequelae**							
**None**	8/9 (88.9)	29/39 (74.4)	1/1 (100)	4/8 (50.0)	26/36 (72.2)	21/44 (47.7)	0.008
**Hypertrophic scar**	1/9 (11.1)	3/39 (7.7)	0/1 (0)	2/8 (25.0)	2/36 (5.6)	12/44 (27.3)	0.055
**Skin discoloration**	0/9 (0)	6/39 (15.4)	0/1 (0)	0/8 (0)	3/36 (8.3)	4/44 (9.1)	0.663
**Transient facial palsy**	0/9 (0)	1/39 (2.6)	0/1 (0)	2/8 (25.0)	5/36 (13.9)	5/44 (11.4)	0.070
**Permanent facial palsy**	0/9 (0)	1/39 (2.6)	0/1 (0)	0/8 (0)	1/36 (2.8)	4/44 (9.1)	0.153
**Facial palsy (any)**	0/9 (0)	2/39 (5.1)	0/1 (0)	2/8 (25.0)	6/36 (16.7)	9/44 (20.5)	0.017

*Excludes patients with Penn clinical stage grade IV (fistulization) at presentation.
NTM, non-tuberculous mycobacteri
